# Pre-Admission Oral Clonidine to Reduce Severe Pre-Operative Anxiety in Pediatric Patients with Behavioral Disorders: A Case Series

**DOI:** 10.3390/children11030264

**Published:** 2024-02-20

**Authors:** Nicole Verdecchia, Ryan Nelson, Shante White, Franklyn Cladis

**Affiliations:** Department of Anesthesiology, Children’s Hospital of Pittsburgh, University of Pittsburgh Medical Center, Pittsburgh, PA 15224, USA; ryan.nelson@chp.edu (R.N.); cladfp@upmc.edu (F.C.)

**Keywords:** pre-operative anxiety, pediatric anesthesia, oral pre-medication, clonidine

## Abstract

Controlling preoperative anxiety is necessary in pediatric patients to avoid adverse effects such as emergence delirium, behavioral problems, post-traumatic stress disorder, anxiety prior to future procedures, and increased analgesic doses in the recovery room. Some patients, especially ones with behavioral issues, have a difficult time arriving at the hospital. Medications given at home can be helpful. We describe a case series of six patients who received pre-admission oral clonidine prior to arrival to the hospital. The patients were all able to enter the hospital without difficulty and the families reported less anxiety and more cooperation subjectively compared with previous experiences. Transient intraoperative hypotension was a side effect of oral clonidine, with no long-term sequelae.

## 1. Introduction

Preoperative anxiety is common in children, most commonly between ages 1–7 years, undergoing diagnostic and therapeutic procedures under anesthesia, and can lead to significant adverse effects, such as emergence delirium, increased analgesic doses in the recovery room, behavioral problems post-operatively, and post-traumatic stress disorder [1]. Several medication classes have been studied to treat anxiety and enhance cooperation in the pre-operative period, including benzodiazepines, ketamine, and alpha-2 adrenergic agonists. There are advantages and disadvantages to each of these classes [1,2]. Clonidine, an alpha-2 adrenergic agonist, has many beneficial effects in the preoperative setting. In addition to analgesia, clonidine reduces nausea, anesthesia requirements, and emergence delirium. Clonidine also reduces anesthesia induction time, oxygen consumption, and has myocardial protective qualities [3,4]. Recently, it has been shown to be similarly effective to midazolam in reducing postoperative negative behavioral changes [3]. The onset time for oral clonidine is 30–60 min, and the half life is around 12 h. The peak effects are within 60–90 min [4]. It also has an excellent safety profile with a very low incidence of bradycardia when dosed appropriately (less than 1%) [5]. Other side effects include hypotension, although existing data suggest that this clonidine induced hypotension does not lead to any cardiac adverse effects [4]. There is one case report of cardiac arrest in a patient with multiple comorbidities who received three doses of clonidine prior to surgery and developed hypotension and bradycardia on induction of anesthesia [6]. The majority of morbidity with clonidine occurs at doses greater than 10 mcg/kg (micrograms/kilogram), as seen from poison information center data in pediatric patients with clonidine exposure from family members’ prescriptions or self-prescriptions for behavioral disorders [7,8].

Clonidine premedication is typically administered after the child has arrived at the hospital and thirty minutes to one hour prior to induction of anesthesia, in children between the ages of 1–7 years. However, children with heightened anxiety, pre-existing behavioral disorders, or children undergoing repeated procedures may be uncooperative and combative when introduced to foreign or frightening environments. Some of these patients are not only challenging during the preoperative preparation in the hospital, but may also be uncooperative during the pre-admission period. In fact, some parents have reported difficulty bringing their child to the hospital. This may include getting into the car and then getting from the car into the hospital or surgical center. These concerns may persist into older ages of larger children, making it very difficult for caregivers. There are no existing data on the use of pre-admission oral clonidine at home in pediatric patients. This project was approved by the University of Pittsburgh quality review committee, and written consent was waived. 

## 2. Case Reports

Pre-hospital oral clonidine was prescribed for six patients with ages ranging from 7–24 years that have been diagnosed with various forms of anxiety, developmental delay, autism spectrum disorder, attention deficit hyperactive disorder, and behavioral concerns. These children all had prior experiences where it was very difficult to get them into the hospital and through the preoperative process, and many of them had a history of being violent or combative.

Patients were identified during a pre-admission evaluation conducted by the Adaptive Care Team. This team consists of pre-operative nurses, child life specialists, and anesthesiologists. Patients that were identified as having severe anxiety, behavioral disorders and/or significant cognitive delay were brought to the attention of three anesthesiologists that serve on this committee. One of the three anesthesiologists contacted the family and discussed the concerns that had been flagged. A multimodal plan was then developed in conjunction with the pre-operative nurses and child life. This included environmental modifications to reduce triggers for aggressive behavior or heightened anxiety. Some of these interventions included an earlier arrival time, skipping the registration area or the vital signs room, and going straight to a quiet private room. In addition to environmental interventions, pharmacologic interventions including pre-admission administration of oral clonidine were discussed. Inclusion criteria for pre-admission clonidine included an ability to take oral medications and currently not taking an alpha-2 agonist. Clonidine was prescribed by one of the three anesthesiologists and families were instructed to administer the medication 1 h prior to arrival at the hospital on the day of the procedure/surgery.

The dose of oral clonidine ranged from 4–7 mcg/kg, with the maximum dose of 600 mcg (Table 1). Four of the six patients received additional premedication when they arrived at the hospital during the preoperative phase (Table 2). None of the patients required any additional medications in the recovery unit, including opioids. Four patients received additional dexmedetomidine in the operating room prior to emergence (Table 2). None of the patients spent a prolonged period of time in the recovery room, and there was one unplanned admission for aspiration on induction requiring an intensive care unit admission (Table 2). All of the patients developed hypotension at some time during their operating room course, two of which occurred on the first blood pressure reading in the operating room, and the other four occurred after the first reading, sometime after the induction of anesthesia. Two patients required pressor medication. The rest of the patients’ hypotension resolved with reduction of the inhalation agent and fluid bolus. Heart rate and oxygenation were normal for all six patients (Table 3). 

## 3. Discussion

Pre-medication in the hospital is effective for preoperative anxiety. Clonidine in particular has been studied in pediatric patients, and has been shown to provide dose-dependent sedation, as well as better quality of separation and mask acceptance, with minimal side effects [9]. However, there were minimal data on medication administration at home prior to arrival. Additionally, patients with pre-existing behavioral disorders tend to be excluded from studies. However, patients with behavioral disorders often have severe preoperative anxiety, leading to difficulties for caregivers in getting the patients into the hospital. Giving sedative medications like benzodiazepines at home carries the risk of airway obstruction, over-sedation, and respiratory depression when co-administered with perioperative opioids, especially in children with pre-existing diseases and syndromes. Clonidine is an effective pre-medication that may exhibit fewer respiratory concerns [1,2,3]. This is the first reported case series of pediatric patients receiving clonidine as a pre-admission premedication for the treatment of severe perioperative anxiety and/or behavioral concerns. 

All families reported improvement in the pre-admission and pre-operative experience compared with their subjective previous experiences. The pre-admission experience was improved because of enhanced cooperation during transport from home and entrance to the hospital. The pre-operative experience was also enhanced because of increased cooperation and sedation. Several families reported that the medication was easy to administer and that they would like to use clonidine for future admissions. The behavioral benefits of clonidine may extend into the postoperative period. Families reported improved behavioral outcomes in the recovery areas without delaying discharge. 

Hemodynamic concerns were a primary concern when this program started. Each patient had blood pressure within the normal range for age upon admission to the hospital, but all experienced hypotension in the operating room. This suggests that premedication with clonidine may predispose patients to hypotension intra-operatively after the co-administration of additional pre-medications and anesthesia induction agents. These hemodynamic changes are manageable with intravenous fluids and vasoactive agents, with no long-term deleterious effects. One patient had an aspiration event, but pre-operative clonidine was not thought to be a contributing factor. This patient had multiple medical comorbidities, including gastroesophageal reflux and seizure disorder. The aspiration occurred after the induction of general anesthesia, which is the most common point for aspiration events during anesthesia when the patient is deeply anesthetized with no airway reflexes. The patient recovered and was extubated the next day. In this case series, all of the families described facilitated entrance to the hospital and enhanced cooperation during the pre-admission and pre-operative phases of patient care. This case series demonstrates that premedication with clonidine prior to hospital arrival is an option for reducing anxiety, enhancing cooperation, and improving the preoperative experience. Hypotension is an important consequence, and clinicians should be prepared to administer fluids or vasopressors. Further experience with pre-admission medication administration will be essential to clarify the value of this process in pediatric perioperative care.

## Figures and Tables

**Table 1:** Demographic data of each patient, their diagnosis and reason for receiving premedication, and clonidine dosing.

Patient #	Age (Years)	Weight (kg)	Diagnoses	Type of Surgery	Home Medications	Reason for Clonidine	Dose of Oral Clonidine (mcg)	Did Clonidine Facilitate Entrance to the Hospital?
1	12	75	Autism, anxiety, attention-hyperactivity deficit disorder, sensory processing disorder	Urology	norethindrone, diphenhydramine, melatonin, sertraline	Extreme anxiety, combative	400	Yes
2	9	50	Anxiety, attention-hyperactivity deficit disorder	Ear, nose, throat surgery, gastrointestinal procedures	escitalopram cetirizine	Extreme anxiety, believes doctors are “going to kill him”	300	Yes
3	7	27	Autism, nonverbal	Ear, nose, throat surgery	melatonin	Uncooperative, kicking, biting	200	Yes
4	13	99	Autism, epilepsy, obesity, developmental delay, speech delay, nonverbal	Dental procedures	oxcarbazepine, lamotrigine, ondansetron	Parental concern, unable to tolerate venous catheter placement	400	Yes
5	24	86	Attention-hyperactivity deficit disorder, anxiety, developmental delay	Dental procedures	omeprazole, famotidine, buproprion, escitalopram, trazodone	Agitation, spits out versed, parental concern of post-op agitation, “wild”	600	Yes
6	10	58	Seizure disorder, cerebral palsy, defiant behavioral problems, anxiety	Gastrointestinal procedures	famotidine, oxcarbazepine, dicyclomine, pantoprazole	Extreme anxiety and uncooperative, combative for mask induction	300	Yes

**Table 2:** Preoperative, intraoperative, and post-operative medications and details.

Patient #	Pre-Induction Midazolam (mg *)	Pre-Induction Ketamine (mg *)	Pre-Induction Dexmedetomidine (mcg)	Intra-Operative Vasopressors	PACU * Opioids	Dexmedetomidine Given after Emergence	Dexmedetomidine Given in OR * (mcg)	Unplanned Admission (Yes/No)	Length of PACU * Stay (Minutes)
1	20 oral	No	no	ephedrine 10 mg	none	no	20	No	49
2	20 oral	500 oral	no	no	none	no	0	No	85
3	None	No	no	no	none	no	12	No	39
4	None	no	no	no	none	no	40	No	71
5	7.5 intramuscular	no	150 intramuscular	150 mcg phenylephrine, 10 mcg epinephrine	none	no	0	Yes	Went to intensive care unit
6	4 intravenous	no	20 intravenous	no	none	no	20	No	54

* mg—milligrams. * PACU—post-anesthesia recovery unit. * OR—operating room.

**Table 3:** Vital signs throughout the course of each patient’s hospitalization. If not recorded, the patient did not cooperate to determine these vital signs. Patient #5 had an aspiration event in the operating room unrelated to clonidine administration, and was transferred to the intensive care unit directly from the operating room.

Patient #	Admission SpO_2_ *	Admission HR *	Admission BP *	1st OR * SpO_2_ *	1st OR * HR *	1st OR * BP *	PACU * SpO_2_ *	PACU * HR *	PACU * BP *	Lowest BP * OR *	Lowest HR * OR *	Lowest BP * PACU *	Lowest HR * PACU*
1	Not recorded	76	Not recorded	98	86	98/54	98	91	119/56	54/26	82	101/54	80
2	98	92	97/20	99	106	55/31	98	86	84/41	55/31	98	84/81	79
3	Not recorded	Not recorded	Not recorded	100	70	66/30	100	59	102/53	66/30	58	100/53	55
4	98	100	102/60	99	80	96/50	98	71	128/76	77/42	68	128/76	69
5	97	88	148/78	92	74	121/69	Went to intensive care unit			60/34	62	Went to intensive care unit	
6	97	104	106/68	100	90	102/53	100	68	103/53	81/38	64	100/51	68

* SpO_2_—pulse oximeter oxygen saturation. * HR—heart rate. * BP—blood pressure. * OR—operating room. * PACU—post anesthesia recovery unit.

## Data Availability

The data presented in this study are available in article.
